# Synthesis of High-Value Bio-Based Polyamide 12,36 Microcellular Foams with Excellent Dimensional Stability and Shape Recovery Properties

**DOI:** 10.3390/polym16010159

**Published:** 2024-01-04

**Authors:** Chin-Wen Chen, Palraj Ranganathan, Bhuvanenthiran Mutharani, Jia-Wei Shiu, Syang-Peng Rwei, Yen-Hsiang Chang, Fang-Chyou Chiu

**Affiliations:** 1 Department of Molecular Science and Engineering, Institute of Organic and Polymeric Materials, Research and Development Center of Smart Textile Technology, National Taipei University of Technology, Taipei 10608, Taiwan; cwchen@ntut.edu.tw (C.-W.C.); rangapalraj@gmail.com (P.R.); bowenshiu@ntut.edu.tw (J.-W.S.); 2 Department of Chemical and Materials Engineering, Chang Gung University, Taoyuan 333, Taiwan; mutharani@gmail.com; 3 Department of General Dentistry, Chang Gung Memorial Hospital, Taoyuan 333, Taiwan; cyh4714@hotmail.com

**Keywords:** bio-based, polyamide, foaming, dimensional stability, shape recovery

## Abstract

The search for alternatives to petroleum-based thermoplastic polyamide elastomers (TPAEs) has recently drawn great interest. In this study, a bio-massed TPAE, PA12,36, was synthesized using 1,12-dodecanediamine (DDA) and fatty dimer acid (FDA, Pripol^TM^1009) precursors via catalyst and solvent-free melt polycondensation. The molecular structure and molecular weight of the PA12,36 were characterized by ^1^H NMR, FTIR, and GPC. PA12,36 displayed a low melting temperature of 85.8 °C, an initial degradation temperature of 425 °C, and a glass-transition temperature of 30.4 °C, whereas it sustained satisfactory tensile strength (10.0 MPa) and superior strain at break (1378%). Furthermore, PA12,36 was foamed by supercritical CO_2_, and the cell size, cell density, and porosity were determined. The entangled long-chained FDA component generated a physically crosslinked network, which promoted the melt viscosity of PA12,36 against elongations of foam cell growth and increased foamability significantly. As a result, uniform structured cellular foams with a cell diameter of 15–24 µm and high cell density (10^11^ cells/cm^3^–10^12^ cells/cm^3^) were successfully achieved. The foaming window was widened from 76 to 81 °C, and the expansion ratio was increased from 4.8 to 9.6. Additionally, PA12,36 foam with a physically crosslinked structure presented a better creep shape recovery percentage (92–97.9%) and sturdier dimensional stability. This bio-based PA12,36 foam is a promising candidate to replace petroleum-based thermoplastic elastomer foams for engineering applications, particularly shoe soles.

## 1. Introduction

Polymer foams, as a sort of lightweight plastic product, cover all sectors of life and industrial manufacture owing to their outstanding attributes, comprising low costs, higher cushioning performance, excellent noise reduction, heat insulation, shock dissipation, and great specific strength. Due to these characteristics, they are used worldwide in numerous areas, such as construction, impact resistance, logistics packing, aerospace, and transport materials [1,2,3]. The growth and utilization of polymer foams can considerably improve energy efficacy and diminish energy consumption, contributing greatly to carbon neutrality and peaking [4,5,6].

Currently, thermoplastic polyamide elastomer (TPE) foams are emerging as candidates in the footwear industry. Polyamide, also known as PA, is a noteworthy semi-crystalline thermoplastic engineering plastic, and is extensively utilized in fiber textiles, automobile engineering, electronics, electrical components, and other industries, owing to its satisfactory flexibility, superior mechanical strength, resistance, and self-lubrication [7,8,9]. PA foams coalesce the benefits of both foaming products and PA; consequently, they have drawn emerging interest in academic research and industrial use in recent years [10,11]. General techniques for fabricating PA foams comprise extrusion foaming, injection molding foaming, bead foaming, and microcellular supercritical carbon dioxide foaming (scCO_2_). Among these foaming techniques, based on the need for ecological safety, PA foams made using the scCO_2_ foaming method are encouraged [12,13]. Compared with conventional blowing agents such as chlorofluorocarbons and alkanes, scCO_2_ is green, inflammable, nonhazardous, and solvable in polymer matrix [14]. Moreover, PA foams achieved by the scCO_2_ foaming method can benefit from polymer weight reduction and material savings, and can offer advantages such as improved flexibility, thermal insulation, elasticity, and energy absorption [15,16,17]. Because of this, more recently, thermoplastic microcellular PA foam has garnered prodigious interest for its utility in the footwear production industry [18]. It circumvents the drawbacks of other elastomer foams, such as the great density of thermoplastic polyurethane (TPU) [19] and inferior durability of ethylene-vinyl acetate (EVA) [20], owing to its distinctive soft- and hard-segment architecture.

Presently, PA12-based thermoplastic PA elastomer (TPAE) is the best known TPAE for footwear foams, namely PEBAX. The exorbitant costs of TPAE foams only apply to high-end footwear [21]. Among TPAE foams, PA6-based foam offers the substantial possibility of large-scale uses owing to its broad raw material sources [22]. However, traditional PA6 or other PAs generally comprise linear networks, poor melt strength, and low viscosity, which are insufficient to preserve foam cell texture and ultimately result in foam cell rupture [22]. Moreover, these features limit its uses in foaming operations, where strain deformity is common. General approaches used to improve PA melt strength comprise nanofiller blending, chain extension, and chemical crosslinking [23,24,25]. However, owing to fillers’ large surface area, melt blending of polymers with nanofillers mostly creates accumulation, especially in high filler loading conditions, making it complex to disperse homogeneously in the polymer system [26]. The effect of extending chains to toughen the material is minimal and thus cannot prevent bubble deformation, and polymers altered by chemical crosslinking may become gelled and nonrecyclable, leading to environmental pollution and resource waste [22].

In addition, large footwear manufacturers using PA foams increase PA waste [27]. The shoe industry is a huge and rapidly developing industry [28]. According to statistics, the worldwide footwear market was evaluated to be worth 373 billion USD in 2021, and that value is set to reach 430 billion USD in 2030 [29,30]. With the rapid development of the shoe industry, polymeric foam waste has also increased considerably [31]. Traditionally, shoe foams are made from fossil-based resources [32]. Developing thermoplastic PA foams from bio-based and ecofriendly feedstock without the use of exogenous additives (crosslinkers, chain extenders, and fillers) is challenging and, due to sustainability and other ecological concerns, is critical to their development. Based on the above consideration, we hypothesize that the synthesis of PA is advantageous using a vegetable oil-based fatty acid monomer with a branched structure. This fatty acid monomer has a long- branched, hanging-chain structure, which can provide the PA with high viscosity, modulus, a crosslink-like structure, and low foaming temperature, which is expected to significantly improve melt viscosity and foamability. Plant oils are an especially promising substitute raw material for the preparation of PA because they are degradable, obtainable in huge quantities, and relatively inexpensive [33]. Noteworthy literature on the manufacture of PAs from vegetable oil is readily obtainable [34]. However, the foaming of PA derived from bio-based fatty dimer acid (FDA) and 1,12-dodecane diamine (DDA), and its potential application, have not yet been studied.

This work aims to solve the cell collapse, extreme volume shrinkage, cell rupture, and poor recovery of PA-foamed materials resulting from their low melt strength, low linear molecular chain stiffness, and low elasticity. In this study, we synthesized a fully bio-based thermoplastic polyamide elastomer (TPAE), PA12,36, using DDA diamine and FDA (Pripol^TM^ 1009) monomers to improve melt strength without the contribution of chemical crosslinkers, by simple melt polymerization. The PA12,36 was comprehensively analyzed in terms of its molecular structure, thermal attributes, and mechanical attributes. In this study, PA12,36 microcellular foams were produced utilizing green scCO_2_. The low melting temperature and melting enthalpy indicated less crystallinity of PA12,36, which was favorable for improving gas permeability and reducing the effect of pressure variance inside and outside the cell on foam contraction. FDA monomers formed physical crosslinks such as chain entanglement, hydrogen, and/or van der Waals bonding, owing to the great number of nonpolar and polar side chains, which increased the foaming melt strength, creep recovery, and structural stability. This approach could reduce footwear pollution and advance the development of bioresource-based PA foams.

## 2. Experimental Section

### 2.1. Materials

Tetrahydrofuran (THF, HPLC grade, 99.9%), 1,12-dodecanediamine (DDA), and chloroform-d (≥99.8%, CDCl_3_) were purchased from Sigma Aldrich. Fatty dimer diacid (FDA, Pripol^TM^ 1009; molecular weight ~570; 100% renewable carbon content; ~1.0% tricarboxylic acid and ~98.7% dicarboxylic acid; iodine value 4.5 g/100 g and acid value 196 mg·g^−1^) were procured from Croda International Ltd. (The Netherlands, Gouda city).

### 2.2. Synthesis of PA12,36

Wholly renewable PA12,36 was synthesized via one-step melt polycondensation in a 2L polymerization reactor equipped with a mechanical stirrer, vacuum pump, nitrogen gas inlet, and reflux condenser. FDA (1000 mmol) and DDA (1010 mmol) were introduced into the reactor and mixed at 50 °C for 30 min. Subsequently, the reaction temperature was augmented by 25 °C every 20 min until attaining 250 °C, to avert unnecessary foaming or bubbling. The polymerization was performed at 250 °C for 12 h and further under 0.7 kPa reduced pressure until the rod climbing phenomenon occurred. After polymerization, the product was poured into ice-cold water. After cooling it to ambient temperature, a transparent PA12,36 was obtained.

### 2.3. Foaming Process

First, PA12,36 specimens for foaming experiment were prepared using a hot-pressing method. The PA12,36 powder was placed in a metal plate, which was placed in a hot-press instrument and heated to 110 °C for 15 min to melt the powder. Afterwards, pressure was applied using a force of 6.3 T. After 15 min of molding, the sample was permitted to cool to 30 °C, then detached from the mold and immediately cut into small pieces of ~5 cm in area. PA12,36 foamed specimens were fabricated using a batch foaming technique employing scCO_2_ as the green-blowing agent. The PA12,36 specimens (~5 cm area) were then placed in a high-pressure vessel. After attaining the needed saturation pressure (S_P_), the foaming system was heated to different temperatures (T_foaming_s) of 70 °C, 76 °C, 79 °C, 81 °C, and 84 °C for 90 min to ensure that all the specimens were completely saturated with scCO_2_. Eventually, PA12,36 foaming was stimulated through rapid depressurization.

### 2.4. Characterization

The comprehensive instrumentation and analysis techniques (FTIR, ^1^H NMR, TGA, DSC, tensile properties, DMA, SEM, cell density, expansion ratio, and creep recovery measurements) are described in the Supporting Information.

## 3. Results and Discussion

### 3.1. Structure Characterization

**Scheme 1:**
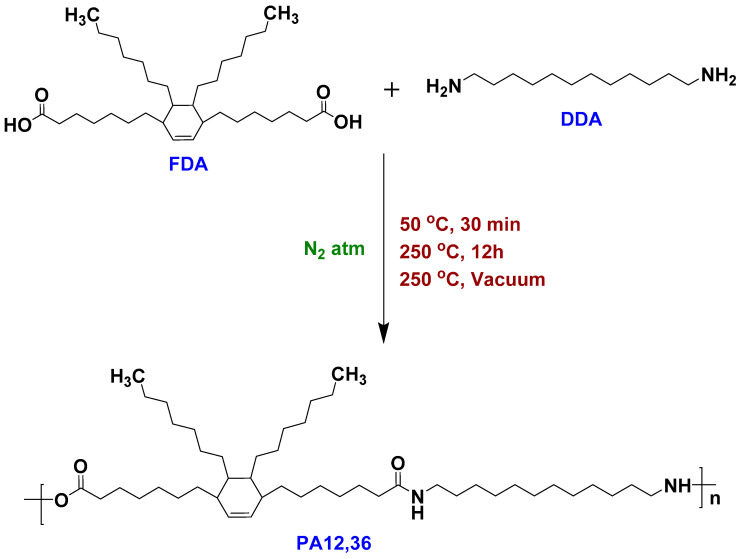
Synthesis of PA12,36 from the polycondensation of DDA and FDA.

**Figure 1:**
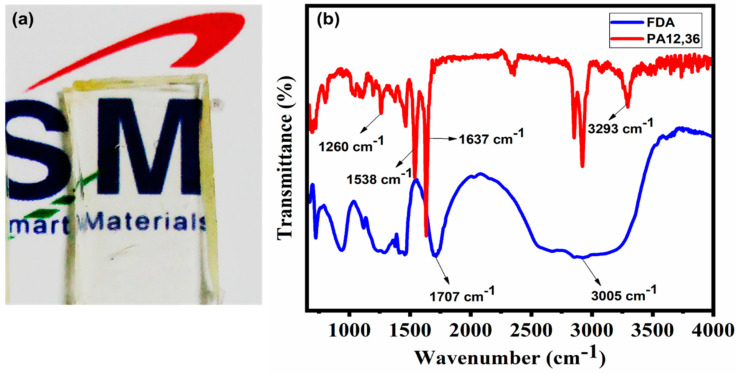
(**a**) Photograph of the synthesized, compression-molded, transparent PA12,36 and (**b**) FTIR spectrographs of FDA and PA12,36.

The primary objective of this study was to synthesize and characterize bio-massed PA12,36 elastomer from renewable monomers and utilize them for foaming applications. PA12,36 was synthesized successfully via simple one-step catalyst and solvent-free melt-polycondensation of renewable FDA and excessive DDA (Scheme 1). PA12,36 is a faint yellowish, transparent, and flexible solid (Figure 1a). PA12,36 films show an excellent transmission of 88.9%, are in the UV region of 500 nm, and their cut-off wavelength (λ_0_ [nm]) is formed at 278 nm (Figure S1). The molecular structure of PA12,36 was confirmed by FTIR and ^1^H NMR spectroscopy. The FTIR spectrograph of PA12,36 (Figure 1b) shows characteristic amide bands at 3293 cm^−1^ (amide-I, N–H vibration), 1637 cm^−1^ (amide II, C=O stretching), 1538 cm^−1^ (amide III, N–H deformation coupled with C–N), and 1260 cm^−1^ (amide IV, N–H bending), created by the reaction of DDA with FDA. Furthermore, the vanishing IR signals at 3005 cm^−1^ and 1707 cm^−1^ indicate that all COOH groups of FDA reacted with DDA amine groups [35].

**Figure 2:**
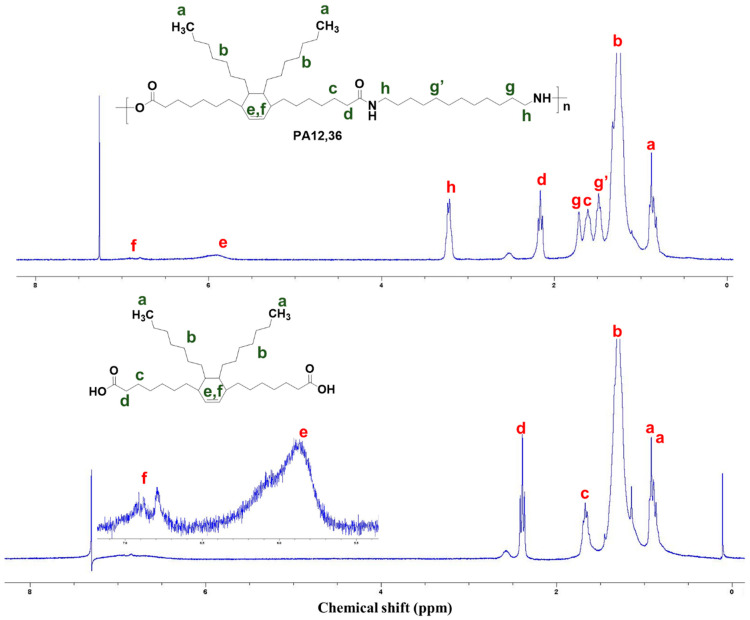
^1^H NMR spectrographs of FDA and PA12,36.

Originally, we analyzed the FDA ^1^H NMR spectrum, which was highly suitable for the comparative examination of the resulting PA12.36 (Figure 2). The proton signals at 0.8–1.0 ppm, 1.2 ppm, 1.6–1.7 ppm, 2.3 ppm, 4.7–5.6 ppm, and 6.6–7.1 ppm were assigned to the dangling –CH_3_ (labeled a), −CH_2_– (labeled b), −CH_2_–CH_2_–COOH (labeled c), −CH_2_–COOH (labeled d), and −CH=CH− (labeled e and f) groups, respectively. The ^1^HNMR spectrograph of PA12,36 clearly revealed the polymerization of DDA with FDA. Originally, the −CH_2_–COOH (2.37ppm) proton signal of the original FDA shifted to 2.17 ppm of the –CONH− groups (labeled d) in PA12,36. This chemical shift was associated with the formation of amide bonds. Additionally, new proton signals at 1.4–1.8 ppm resembled the −CH_2_– protons (labeled g and g’) existing in the DDA unit of PA12,36, while the new generated proton signal at 3.26 ppm belonged to the −CH_2_– group adjacent to the –CONH− groups (labeled h) in PA12,36. In the end, the −CH=CH– protons were still present, the double bonds were not activated during polymerization, and no radical crosslinking ensued amid the molecules [36]. These outcomes sufficiently indicated that the polymerization of FDA with DDA was completed, and PA12,36 was successfully manufactured.

The weight-average molecular weight (M_w_), polydispersity index (Ð), and number-average molecular weight (M_n_) of synthesized PA12,36 were determined by GPC using THF as the solvent. The resulting M_w_, M_n_, and Ð values were 52,900 g mol^−1^, 31,400 g mol^−1^, and 1.6, respectively.

### 3.2. Thermal Properties

**Figure 3:**
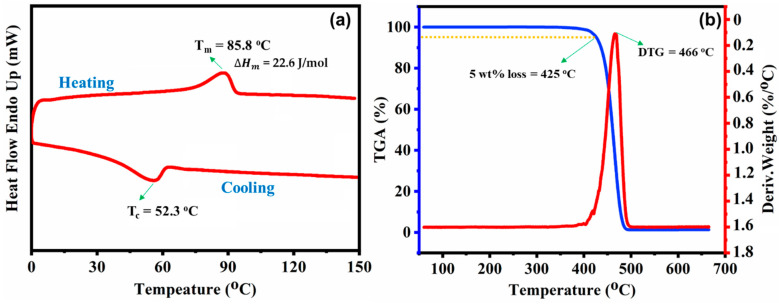
(**a**) The DSC heating and cooling curves and (**b**) TGA (blue) and DTG (red) curves of PA12,36.

Figure 3a displays the DSC heating and cooling thermograms to determine the melting temperature (T_m_) and crystallization temperature (T_c_) of PA12,36. The T_m_ of PA12,36 was recorded to be 85.8 °C and the $\Delta H_{m}$ was 22.6 J/mol. This T_m_ value is lower than the T_m_s of traditional PAs, such as PA10,12 (T_m_ = 190 °C) [37], PA6,10 (T_m_ = 221 °C) [37], and PA6,6 (T_m_ = 262 °C) [38], chiefly due to the lower –CONH– linkage concentration and there being more methylene units in the repeating unit of PA12,36. The crystallinity (X_c_) of PA12,36 was estimated to be ca. 11% with Supplementary Equation (S1). Two aspects influenced the low T_m_ and X_c_ of PA12,36. Primarily, PA12,36 comprised entangled methyl long chains in its repeating unit, which escalated chain disorder and prevented the formation of an ordered crystalline segment. Secondly, amid the ordered and disordered phases, the lengthy repeating network of PA12,36 structured kinetic obstacles of conformational entropy, which were hard to surpass [27]. As shown in the DSC cooling trace in Figure 3a, PA12,36 had an exothermic peak at 52.3 °C, labeled as T_c_.

The thermal stability of PA is crucial in the foaming process and its practical uses. The TGA result of PA12,36 is illustrated in Figure 3b. Unlike traditional Pas, where the initial weight loss owing to the vaporization of water occurs at ca. 100 °C, the decomposition of PA12,36 began at ca. 425 °C (temperature at 5% weight loss). As PA12,36 comprises numerous nonpolar groups and is water repellent in its molecular structure, no degradation signal of water was seen in the TGA trace. The temperature at the maximum weight loss of PA12,36 was recorded from the DTG trace, at 466 °C. The great thermal permanency (468 °C) of PA12,36 supports its foaming process and engineering uses. The high thermal stability of PA12,36 may be due to physical crosslinking, such as chain, hydrogen, and/or van der Waals bonding. The augmented crosslinking networks can decrease the chain segmentation mobility of PA12,36, which can increase its thermal stability.

**Figure 4:**
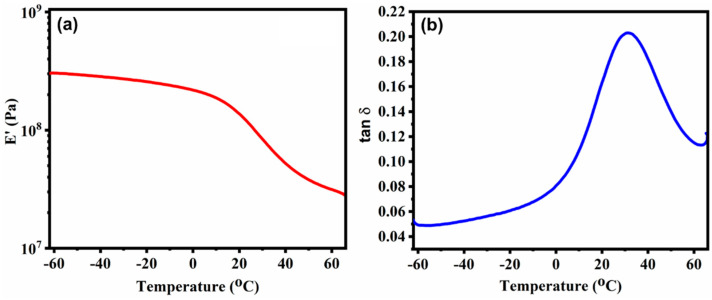
(**a**) storage modulus (*E*′) and (**b**) tan *δ* trace of PA12,36.

The DMA traces of PA12,36 are displayed in Figure 4. The storage modulus (*G*′) indicates the elastic behavior of PA12,36, and is connected to segmental movement. In the *G*′ curve of PA12,36, the glass-to-rubber shift region began at −4.4 °C, and *G*′ decreased gradually with increasing temperature (Figure 4a). Subsequently, before attaining the liquid phase, PA12,36 underwent a narrow rubbery plateau at ca. 53.1 °C. The tan δ signal (Figure 4b) shows the molecular mobility (viscoelasticity) of PA12,36, and can be used to assess glass-transition temperature (T_g_). The PA12,36 elastomer showed a T_g_ value of 30.4 °C, higher than that of PA6,36, PA2,36, PA4,36, and PA9,36 [27,36]. FDA comprises an aliphatic ring and a low –CONH– linkage concentration, which augment the degree of conformational free-volume disarray and subsequently promote molecular chain movement, accounting for the T_g_ tendency. These results are in good agreement with prior reports [39].

### 3.3. Tensile Properties

**Figure 5:**
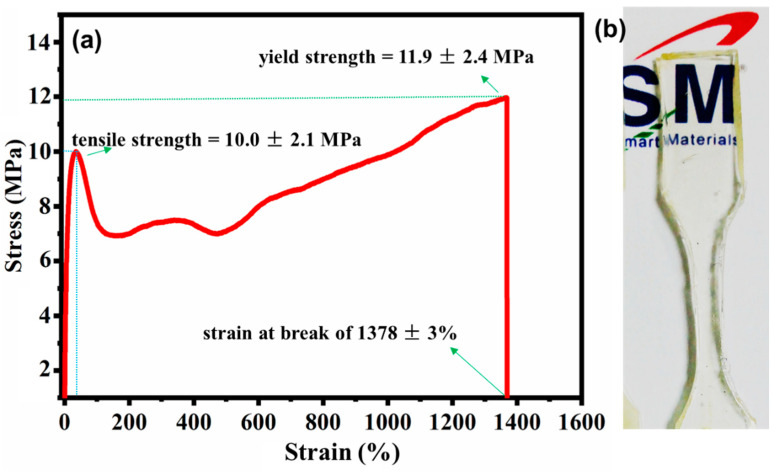
(**a**) Tensile s-s curve and (**b**) photograph of PA12,36 tensile test specimen.

The stress-strain (s-s) trace of PA12,36, illustrated in Figure 5a, has a curve resembling that of TPEs comprising PA components [39]. The tensile curvature is not very smooth, possibly owing to driftage in chain entanglements during elongating. Comparable s-s curves have been formerly documented for a few other PAs, such as PA6,20, PA6,16, PA4,36, and PA6,36 [27,40,41]. PA12,36 had a yield strength of 11.9 ± 2.4 MPa, tensile strength of 10.0 ± 2.1 MPa, and strain at break of 1378 ± 3%. The tensile strength of PA12,36 is inferior to that of PA4,36 due to a lower –CONH– linkage concentration in PA12,36 [27]. The –CONH– linkage concentration affected intermolecular interlinkages and chain rigidity. For instance, H–bonding formation via –CONH– linkages led to a reduction in elongation at break and increased tensile strength.

### 3.4. Foaming Performance

After analyzing structural, thermal, and mechanical attributes, the foamability of bio-based PA12,36 elastomer was further explored. A green scCO_2_ batch foaming technique was implemented in this work, and it entailed two steps: (i) absorption of scCO_2_ into the PA matrix in a supercritical fluid phase, and (ii) development of cells using nucleation and a saturation pressure release procedure. Several factors, such as saturation temperature (T_foaming_), saturation pressure (S_pressure_), and foaming time (F_time_) could affect the foaming performance. The T_foaming_ setting is a pivotal factor in the development of foam structure. T variance in T_foaming_ and T_m_ determines the polymer’s crystalline structure, which could directly influence the stability of foam’s cellular texture and the growth and nucleation mechanisms of bubbles in the scCO_2_ foaming operation.

**Figure 6:**
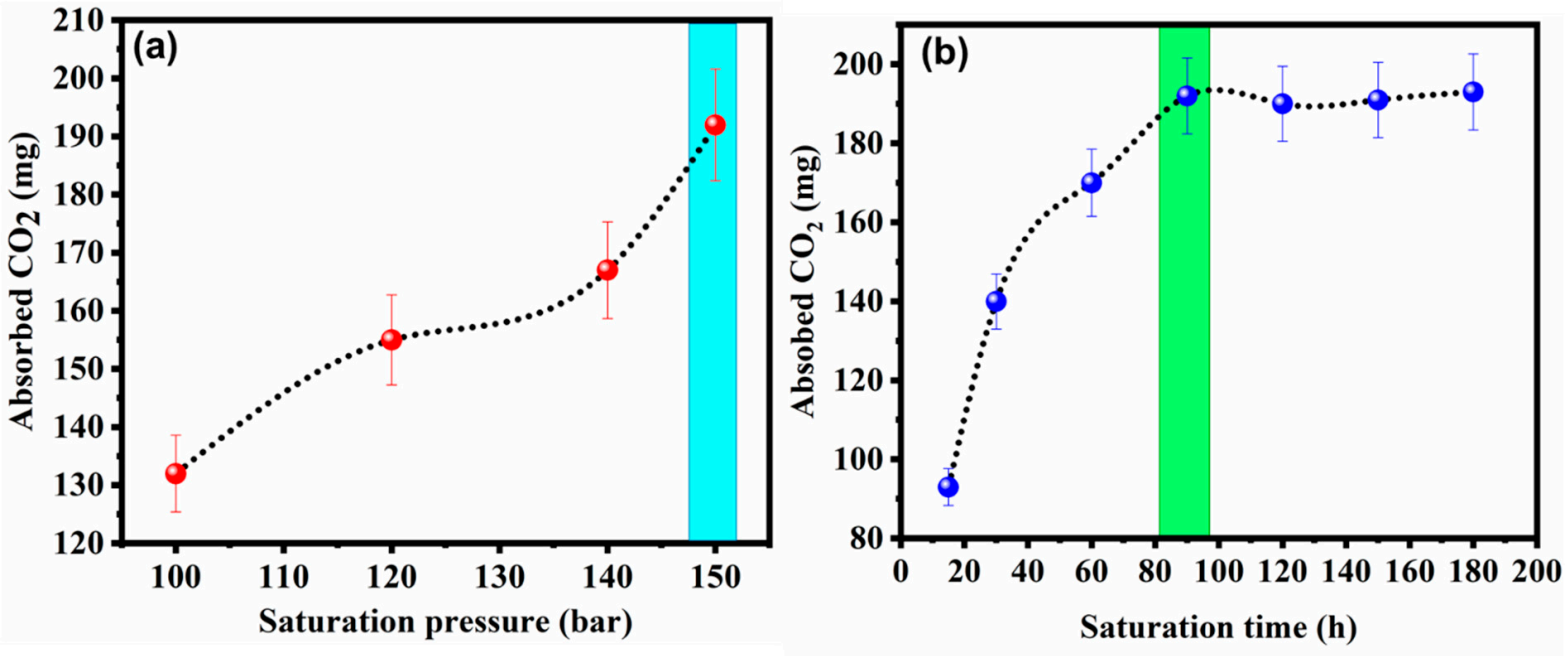
(**a**) CO_2_ absorption of PA12,36 at different S_pressure_s and (**b**) CO_2_ absorption of PA12,36 at 79 °C.

To find the optimal S_pressure_ and F_time_ conditions for the scCO_2_ foaming process, we first investigated the CO_2_ absorption properties in terms of S_pressure_ and F_time_. The measurement procedure for CO_2_ absorption has been explained in detail in our previous research [42]. The CO_2_ absorption of PA12,36 upsurged as the applied S_pressure_ increased, and reached a peak at 150 bar (at T_foaming_ = 79 °C; F_time_ = 90 min), as displayed in Figure 6a, which demonstrated that an S_pressure_ of 150 bar was suitable for CO_2_ absorption and foaming attributes. The interaction between amide groups in PA12,36 and oxygen groups in CO_2_ resulted in a plasticization effect and H-bonds, which augmented the movement of PA12,36 networks and absorbed more CO_2_ [43]. In general, polymeric foams produced at higher S_pressure_ would have smaller cell sizes because more CO_2_ is dissolved in the polymer matrix, which affords greater driving energy for cell nucleation compared with lower S_pressure_ and results in more nuclei [44]. In Figure 6b, the CO_2_ absorption tendency plotted against the saturated F_time_ of PA12,36 specimens under 79 °C and 150 bar conditions is shown. After soaking for 90 min, the CO_2_ absorption of PA12,36 reached a saturation level, so using an F_time_ of 90 min was adequate for this work.

**Figure 7:**
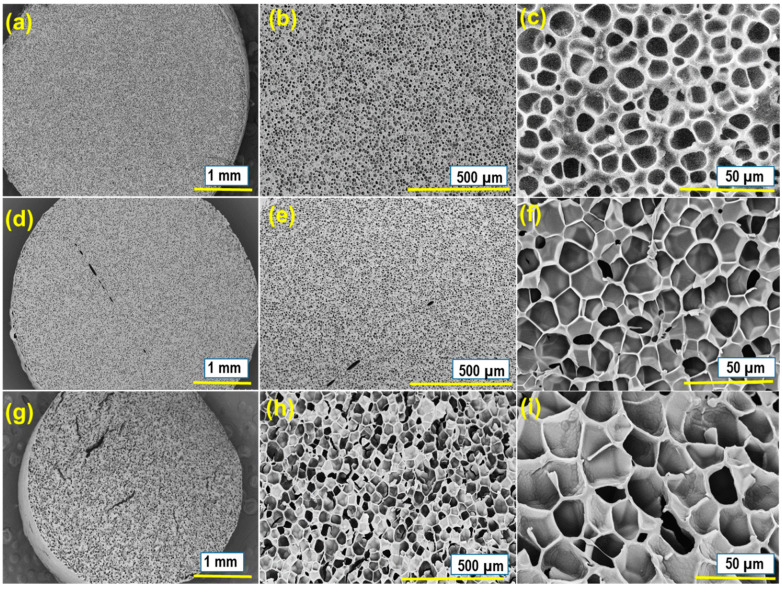
Different magnification SEM cellular micrographs of PA12,36 foams prepared under various T_foaming_s: (**a**–**c**) 76 °C, (**d**–**f**) 79 °C, and (**g**–**i**) 81 °C.

To further validate the above measurements and aid in the development of microcellular foam structure, the PA12,36 specimen was foamed at different T_foaming_s under 150 bar (F_time_ = 90 min). Foaming temperatures of 70 °C, 76 °C, 79 °C, 81 °C, and 84 °C were applied to study the foaming performance. The cellular topography of the PA12,36 foam at diverse T_foaming_s is presented in Figure 7. When the T_foaming_ was 70 °C, the PA12,36 specimen had a poor foam texture with a large nonfoaming area (Figure S2). Furthermore, at an S_pressure_ of 150 bar, the unfoamed region reduced as the T_foaming_ increased from 76 °C to 81 °C, and all foams showed uniform cell textures (Figure 7a–i). Under constant S_pressure_ (150 bar), when the T_foaming_ was comparatively low (i.e., 70 °C), numerous unmelted hard-phase crystals led to high melt strength and were thought to be a major barrier to the foam cell growth of PA12,36, as displayed in Figure S2. With an escalation in T_foaming_, the melt strength of PA12,36 decreased, thereby reducing the barrier to nucleation and growth of foam cells. Also, when the T_foaming_ was 83 °C, the foam cells collapsed, as shown in Figure S3. The worsened melt strength of PA12,36 at high T_foaming_ might be a reason for the contraction of foams. The coalescence and rupture of cells and contraction have pivotal impacts on the cellular texture of PA foams, enlarging cells, reducing density and surface area, and deteriorating resilience and hardness. Hence, T_foaming_ must be controlled to prevent microcellular material being produced by insufficient foaming temperature, as well as exorbitant cell growth caused by high foaming temperature. Foams prepared at T_foaming_s ranging from 76 °C to 81 °C had a uniform cell texture, indicating that the PA12,36 synthesized in this study had a wide processing window in the scCO_2_ foaming process.

### 3.5. Expansion Ratio (E_R_), Average Cell Diameter (D), and Cell Density

**Figure 8:**
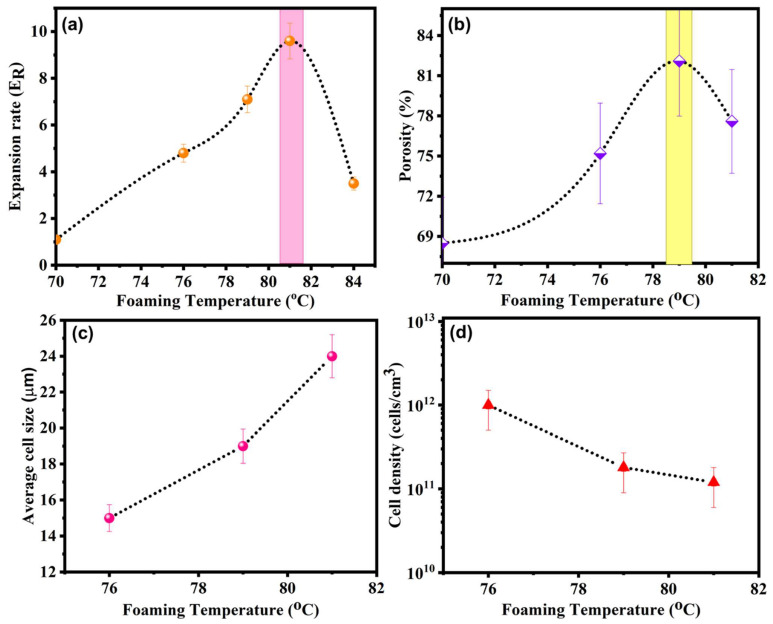
(**a**) E_R_, (**b**) porosity, (**c**) average cell diameter (*D*), and (**d**) cell density of PA12,36 foams prepared at different T_foaming_s (S_pressure_ = 150 bar, F_time_ = 90 min).

The statistical results for the E_R_, *D*, and cell density of prepared foams are shown in Figure 8. As Figure 8a displays, the E_R_ initially increased and then decreased with increasing T_foaming_, creating a characteristic bell curve that is common for microcellular polymer foaming. The maximum E_R_ for PA12,36 foams was reached at a T_foaming_ of 81 °C. An increasing T_foaming_ could decline the melt viscosity of PA12,36, which could greatly limit bubbles’ growth. A higher T_foaming_ can promote the diffusion of CO_2_ and prolong bubble growth duration [45]. Increasing the T_foaming_ further to 84 °C led to excessive foam cell growth due to low melt viscosity, and then caused foam shrinkage. Figure 8b illustrates the porosity of PA12,36 foams as a function of T_foaming_. The porosity of PA12,36 foams foamed at 84 °C cannot be calculated due to foam shrinkage. The porosity of PA12,36 foams initially increased and subsequently decreased with T_foaming_, which was consistent with the changing tendency of E_R_, as shown in Figure 8a.

Figure 8c shows the *D* of PA12,36 foams over various T_foaming_s (S_pressure_ = 150 bar, F_time_ = 90 min). It can be seen that the *D* of PA12,36 foams ranged from 15 to 24 µm, signifying the *D* could be controlled by regulating the T_foaming_ [46]. Figure 8d plots the cell density of PA12,36 foams prepared at different T_foaming_s (S_pressure_ = 150 bar, F_time_ = 90 min). The cell densities of PA12,36 foams attained varied between 10^11^ cells/cm^3^ and 10^12^ cells/cm^3^, mostly within an order of magnitude, demonstrating that the influence of T_foaming_ conditions on cell density was minimal. This might be associated with the high melt viscosity of PA12,36.

### 3.6. Cyclic Creep Recovery and Dimensional Stability

**Figure 9:**
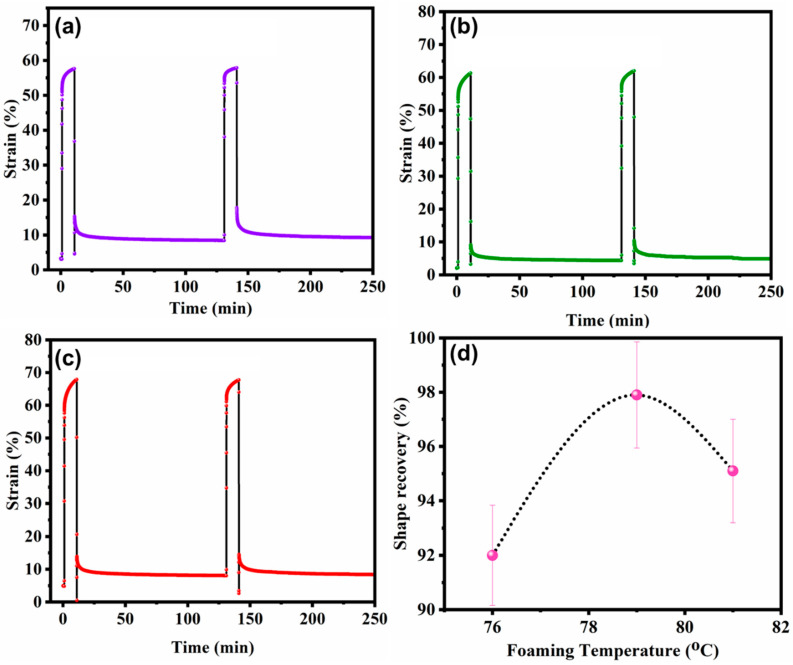
Cyclic creep recovery traces of PA12,36 foams prepared under different T_foaming_s: (**a**) 76 °C, (**b**) 79 °C, and (**c**) 81 °C; (**d**) Creep recovery percentage of PA12,36 foams prepared under different T_foaming_s.

The polymer foam specimens’ dimensional stability under tensile/compression cyclic loading or unloading is pivotal for practical uses, especially in footwear. Therefore, a creep recovery experiment was conducted to further investigate the shape recovery (R_V_ (%)) attributes of PA12,36 foams, as shown in Figure 9a–c, and the foaming temperature-dependent R_V_ (%) was plotted in Figure 9d. PA12,36 foam distorted its shape under force and creep strain rose with time, owing to PA12,36 chain networks stretching. Elastic retrieval of the stretched PA12,36 chains ensued, related to a reduction in strain [47]. However, all of the PA12,36 showed an excellent R_V_ (%); specifically, PA12,36 foamed at 79 °C had an R_V_ of 97.9%. The R_V_ (%) variance between PA12,36 foams foamed at different T_foaming_s might be due to the *D* and E_R_ of the individual foams. As shown clearly in Figure 9, the cyclic creep recovery curves had very little distortion after loading–unloading cycles, indicating the outstanding elasticity of manufactured PA12,36 foams. Importantly, the creep experiment results demonstrated that the fabricated foams were stiffer, sturdier, and more resilient, and had better elasticity.

### 3.7. Dimensional Stability

Shrinkage is common in foam materials. Significant foam shrinkage occurs within seconds or minutes, completely affecting the use of foamed materials. Figure S4 shows the growth of E_R_ and average cell size with time under different T_foaming_s. There were no obvious changes in the expansion rate and average cell size of PA12,36 foams with different times, indicating excellent dimensional stability.

**Figure 10:**
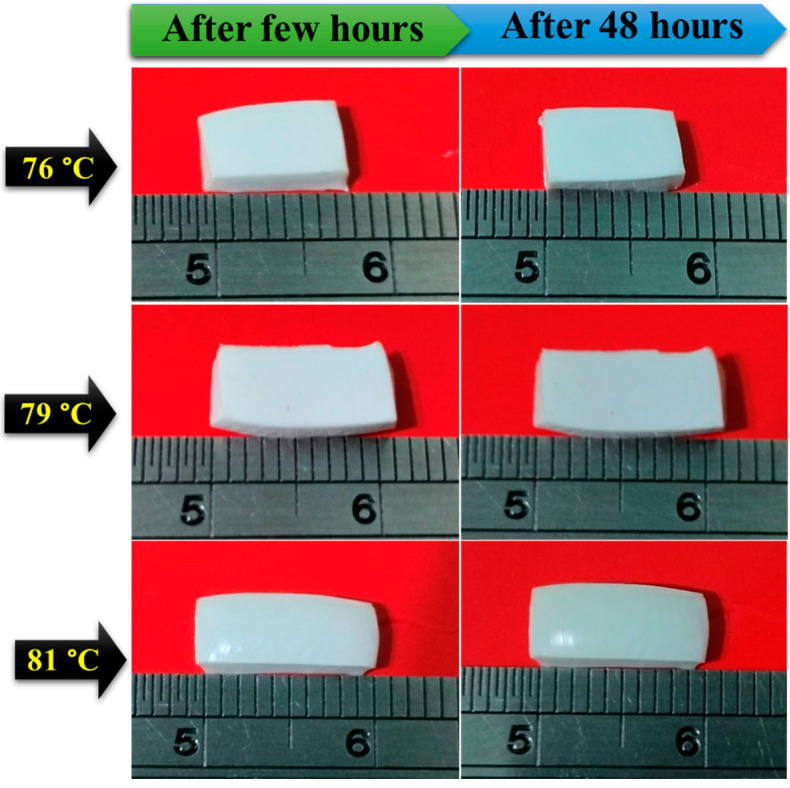
Photographs of PA12,36 foams prepared at different T_faoming_s and the dimensional stability of the foams at different periods.

Therefore, the foams prepared at 73 °C, 76 °C, and 81 °C had good dimensional stability with no shrinkage, which might be attributed to cell structure, scCO_2_ diffusivity, and rheological features. In summary, the phenomenon appearing in Figure S4 indicates that the stability of PA12,36 foam has several benefits. The chain-entanglement network of long-chain branched FDA and the augmented physical crosslink density obstructed PA12,36 chain relaxation and combated foam contraction. Figure 10 shows the dimensional stability behavior of PA12,36 foams recorded by an optical camera. After 24 h of aging, all foam samples foamed under different T_foaming_s had better foam structure stability. Hence, the better structural constancy and foamability of PA12,36 circumvents the foaming complications of inadequate melt viscosity and high crystallinity in traditional PAs.

## 4. Conclusions

A green method was used to synthesize wholly bio-based PA12,36 by catalyst- and solvent-free melt polycondensation using DDA and FDA precursors derived from sustainable resources. The as-prepared PA12,36 showed an M_w_ of 52,900 g mol^−1^, M_n_ of 31,400 g mol^−1^, T_m_ of 85.8 °C with a low X_c_ of 11%, T_g_ of 30.4 °C, and T_d5%_ of 425 °C. The tensile stress and strain at break of PA12,36 were 10.0 ± 2.1 MPa and 1378 ± 3%, respectively. Moreover, PA12,36 with low T_m_ and low X_c_ was foamed using the scCO_2_ method. The as-prepared foams unveiled a narrow and low T_foaming_ window of 73–81 °C. The greatest foam E_R_ of 9.6 was achieved at 76 °C, and the increased T_foaming_ tended to induce foam cell coalescence owing to the inferior melt viscosity (strength) of PA12,36, resulting in a significant reduction of E_R_ (5.5). Lastly, structure-tunable PA12,36 foams were prepared with an E_R_ of 4.8–9.6, an average cell diameter range of 15–24 μm, and a high cell density of up to 10^11^–10^12^ cells/cm^3^. Moreover, PA12,36 foams showed excellent creep shape recovery performance (92–97.9%) and sturdier dimensional stability. The findings of this study reveal a promising, sustainable way to utilize bio-massed resources in the fabrication of PA foam materials in the polymer industry.

## Data Availability

Data are contained within the article.

## Supplementary Materials

The following supporting information can be downloaded at: https://www.mdpi.com/article/10.3390/polym16010159/s1, Figure S1. UV-Visible spectrum of PA12,36 film, Figure S2. SEM micrographs of PA12,36 foamed at 66 °C (S_P_ = 150 bar, F_time_ = 90 min), Figure S3. SEM micrographs of PA12,36 foamed at 83 °C (S_P_ = 150 bar, F_time_ = 90 min), and Figure S4. (a) Evaluation of (a) E_R_ and (b) average cell size of the PA12,36 foams foamed at different T_foaming_ under time variance: title. Ref. [48] is cited in the Supplementary File.
