# Supplementary material for: Synthesis of High-Value Bio-Based Polyamide 12,36 Microcellular Foams with Excellent Dimensional Stability and Shape Recovery Properties
Source: Polymers (Basel). 2024 Jan 4;16(1):159. doi: 10.3390/polym16010159 (PMC10780462; doi:10.3390/polym16010159)
Supplement: Supplementary file 1 [file polymers-16-00159-s001.zip › polymers-2736142-supplementary.pdf]

Structural Characterizations

The chemical structure of the PA12,36 was confirmed by ^{1}H NMR spectroscopy, which was performed on a Bruker Avance 400 MHz NMR spectrometer using $CDCl_3$ as a solvent (solvent signal of $CDCl_3$ for ^{1}H at 7.26 ppm) at 25 ºC.

FTIR spectra were gained using a Nicolet 5700 FTIR spectrometer (Bruker, Germany). The wavenumber range was 4000 cm^{-1} to 500 cm^{-1} (spectrum resolution; 2 cm^{-1}) and the specimen was scanned in 32 co-added times.

The number-average molecular weight (M_n), polydispersity index (Đ), and weight-average molecular weight (M_w) of all of PA12,36 were evaluated by gel permeation chromatography (GPC, JASCO RI-2031). THF was utilized as an eluent with a 0.75 mL/min flow rate. Polymer samples were prepared at a concentration of 2 mg mL^{-1} in THF eluent. Linear polystyrene was employed as a standard for calibration. A refractive detector index was operated at 25 °C.

DSC traces were gained from the SHIMADZU AGS-X 500N analyzer (Waltham, MA, USA) under an N_2 flow of 20 mL min^{-1}. The sample (5 mg) was first heated from 0 ºC to 150 ºC with 10 ºC min^{-1} ramp, and then the temperature was kept at 150 ºC for 3 min. Then, it subsequently, cooled down to 0 ºC, and hold for 3 min. The melting temperature (T_m), melting entropy (ΔH_m), crystallization temperature (Tc), and crystallization entropy (ΔH_c) were obtained. The degree of crystallinity ($X_{c\%}$) of the PA12,36 was calculated from the DSC test by the following equation.

$$X_{c\%} = \frac{\Delta H_m}{\Delta H_m^o} \, x \, 100\% \qquad \text{(S1)}$$

where ΔH_m is the heat fusion of the sample examined, estimated from a second heating scan, ΔH_m^o is the heat of fusion of PA6,36 (ΔH_m^o = 198 J/mol).

The thermal stability of PA12,36 was determined by TGA and DTG experiment (Q500 TGA analyzer, TA instrument, USA)) in an N_2 atmosphere (40 mL min^{-1} flow rate). The sample was heated from 50 ºC to 650 ºC with a heating rate of 10 ºC min^{-1}.

DMA testing was conducted on a TA Q800 instrument in the temperature range from –65 to 65ºC at a frequency of 1Hz and with a heating rate of 3 ºC min^{-1}. All the TPEE samples had sizes of 10 mm (length) x 0.5 mm (thickness) x 6 mm (width).

The tensile attributes of PA12,36 was obtained at 25 ºC Universal Tensile Testing Machine INSTRON 3800R, according to ASTM D638 standard (Norwood, USA). The testing specimens were fabricated by injection molding and the test was conducted at room temperature with the crosshead-stretching rate of 50 mm.min^{-1}. At least six samples were tested and an average calculated value was provided.

The cellular morphology of PA12,36 foams was performed by XL 30 ESEM FEG scanning electron microscope (SEM) with a working voltage of 12 kV. To confirm complete foam morphology, the foam was frozen in liquid N_2 for ~5 min and promptly cracked. The cross-section of the foam was sputtered with gold to enhance conductivity before SEM observation.

The average cell size was calculated by *Image J@* software based on the SEM morphology. The calculation of expansion rate, cell diameter, and cell density are described in our previous work [S1,S2].

Creep recovery testing

Creep tests were implemented in tensile mode using a DMA850 (TA Instruments, USA). The foams were stretched at a fixed force of 1 MPa to a certain strain for 50 min at 25 °C. Subsequently, the foam's shape recovery was monitored for 50 min after the force was released. The shape recovery was determined using the following Eq. S2, where ε represents the maximum strain at the applied force and ε_f denotes the residual strain after releasing force.

$$R_{\vartheta} = \frac{\varepsilon - \varepsilon_f}{\varepsilon} \quad \text{(S2)}$$

Results and Discussion

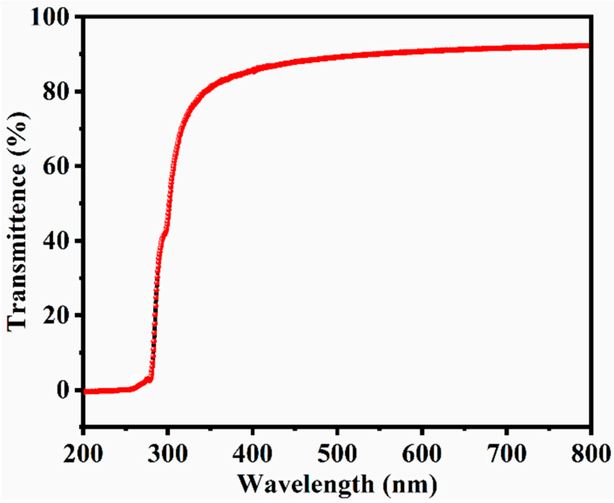

Figure S1. UV-Visible spectrum of PA12,36 film.

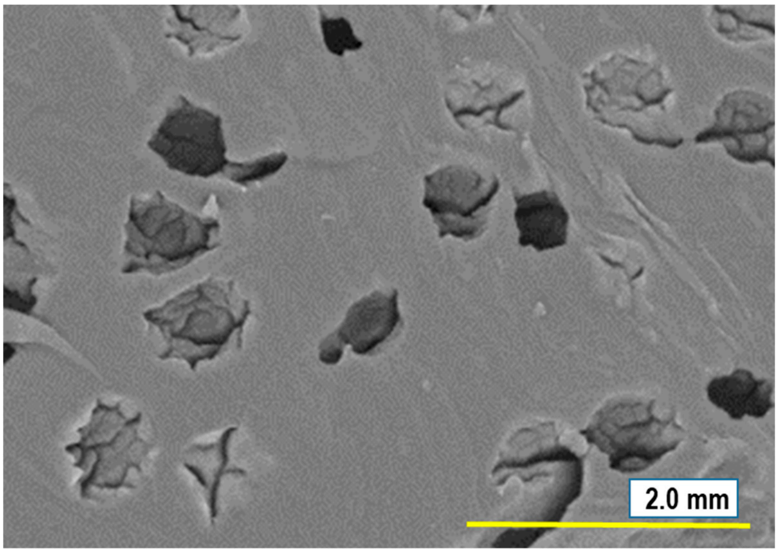

Figure S2. SEM micrographs of PA12,36 foamed at 66 ºC (SP = 150 bar, F_{time} = 90min).

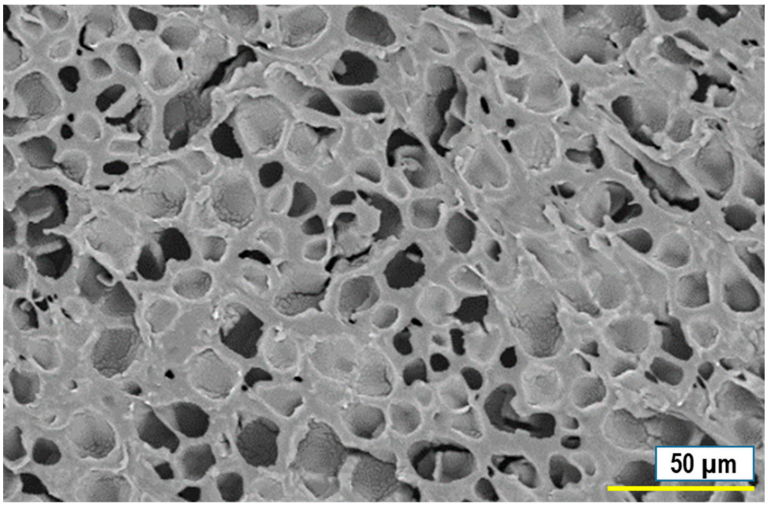

Figure S3. SEM micrographs of PA12,36 foamed at 83 ºC (SP = 150 bar, F_{time} = 90min).

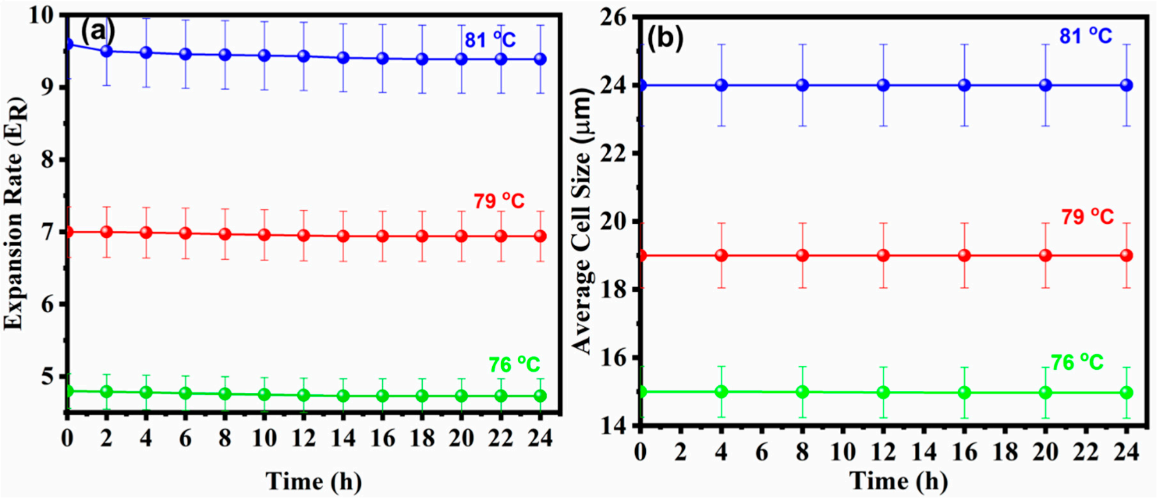

Figure S4. (a) Evaluation of (a) E_R and (b) average cell size of the PA12,36 foams foamed at different $T_{foaming}$ under time variance.

References

S1. Ranganathan, P.; Chen, C.W.; Tasi, M.C.; Rwei, S.P.; Lee, Y.H. Biomass Thermoplastic (Co) polyamide Elastomers Synthesized from a Fatty Dimer Acid: A Sustainable Route toward a New Era of Uniform and Bimodal Foams. *Industrial & Engineering Chemistry Research*, **2021**, 60(33), 12139-12154.

S2. Lee, Y.H.; Lee, C.W.; Chou, C.H.; Lin, C.H.; Chen, Y.H.; Chen, C.W.; Way, T.F.; Rwei, S.P. Sustainable Polyamide Elastomers From a Bio-Based Dimer Diamine for Fabricating Highly Expanded and Facilely Recyclable Microcellular Foams via Supercritical CO_2 Foaming. *European Polymer Journal*, **2021**, 160, p.110765.
